# Unilateral anterior uveitis complicating zoledronic acid therapy in breast cancer

**DOI:** 10.1186/1471-2407-5-156

**Published:** 2005-12-06

**Authors:** Nagi S El Saghir, Zaher K Otrock, Jamal H Bleik

**Affiliations:** 1 Division of Hematology-Oncology, Department of Internal Medicine, American University of Beirut Medical Center, Beirut, Lebanon; 2 Division of Ophthalmology, Rizk Hospital and Lebanese University, Beirut, Lebanon

## Abstract

**Background:**

Zoledronic acid is very widely used in patients with metastatic bone disease and osteoporosis. Only one case of bilateral uveitis was recently reported related to its use.

**Case presentation:**

We report the first case of severe unilateral anterior uveitis in a patient with breast cancer and an intraocular lens. Following zoledronic acid infusion, the patient developed severe and dramatic right eye pain with decreased visual acuity within 24 hours and was found to have a fibrinous anterior uveitis of moderate severity The patient was treated with topical prednisone and atropine eyedrops and recovered slowly over several months.

**Conclusion:**

Internists, oncologists, endocrinologists, and ophtalmologists should be aware of uveitis as a possible complication of zoledronic acid therapy. Patients should be instructed to report immediately to their physicians and treatment with topical prednisone and atropine eyedrops should be instituted immediately at the onset of symptoms. This report documents anterior uveitis as a complication of zoledronic acid therapy. This reaction could be an idiosyncratic one but further research may shed more light on the etiology.

## Background

Bisphosphonates are very widely used the the treatment of metastatic bone disease and osteoporosis. They are structurally analogues to pyrophosphates. They inhibit hydroxyapatite crystal development and dissolution by affecting the ionic makeup of the hydration layer that surrounds the crystals in suspension [1]. They also inhibit osteoclasts. They are very commonly used for osteoporosis and for cancer patients with metastatic bone disease. The most commonly used bisphophonates are etidronate, clodronate, pamidronate, alendronate and more recently zoledronic acid. Zoledronic acid is generally very well tolerated. General side effects of bisphosphonates include transient low-grade fever, fatigue, arthralgia or myalgia, nausea, increased bone pain, fluctuations in serum ion levels (calcium, magnesium, and phosphorus), and occasional elevations in serum creatinine [2].

Severe anterior uveitis has been reported with alendronate [3-5] and pamidronate [6-8]. One recent case report described uveitis as a complication of zoledronic acid in a patient with a normal eye [9]. That patient was reported to have suffered from bilateral anterior uveitis with concurrent bilateral conjunctivitis after 48 hours of administration of zoledronic acid. This is the first case of acute unilateral anterior uveitis in a patient with an intraocular lens.

## Case presentation

A 60-year-old woman was diagnosed to have a left breast cancer stage T_2 _N_1 _M_0 _hormone receptor-positive in 1997. She was treated with modified radical mastectomy, adjuvant chemotherapy with cyclophosphamide, adriamycin and 5-fluorouracil, followed by adjuvant hormonal therapy. She completed five years of adjuvant tamoxifen in 2003 and and was started on letrozole adjuvant therapy in January 2004 [10].

The patient had an ocular history significant for bilateral dacryocystitis, cataracts and open angle glaucoma, left dacryocystectomy (DCR) in 1997, right DCR in 2000 and 2003, as well as a right eye phacoemulsification with implantation of a hydrophilic acrylic intraocular lens in 2003 with a postoperative uncorrected visual acuity of 20/25. The patient was maintained on antiglaucoma treatment in the form of topical beta blocker eyedrops for both eyes, combined with prostaglandin eyedrops for her left eye.

**Figure 1:**
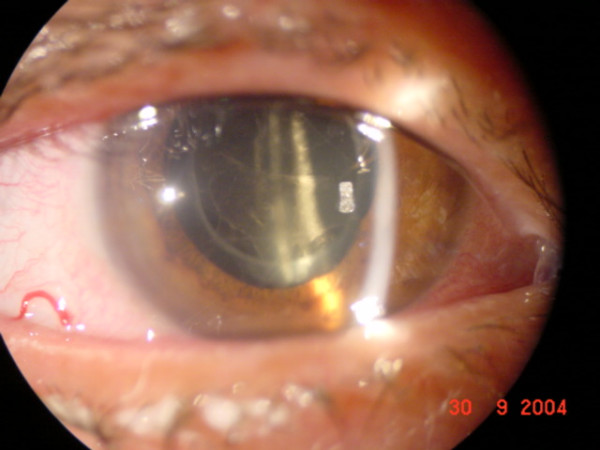
Acute inflammatory fibrinous uveitis: Slit lamp examination showing marked ciliary injection of the right eye, and a 2+ flare and inflammatory reaction in the anterior chamber, with also an inflammatory fibrinous pupillary membrane.

**Figure 2:**
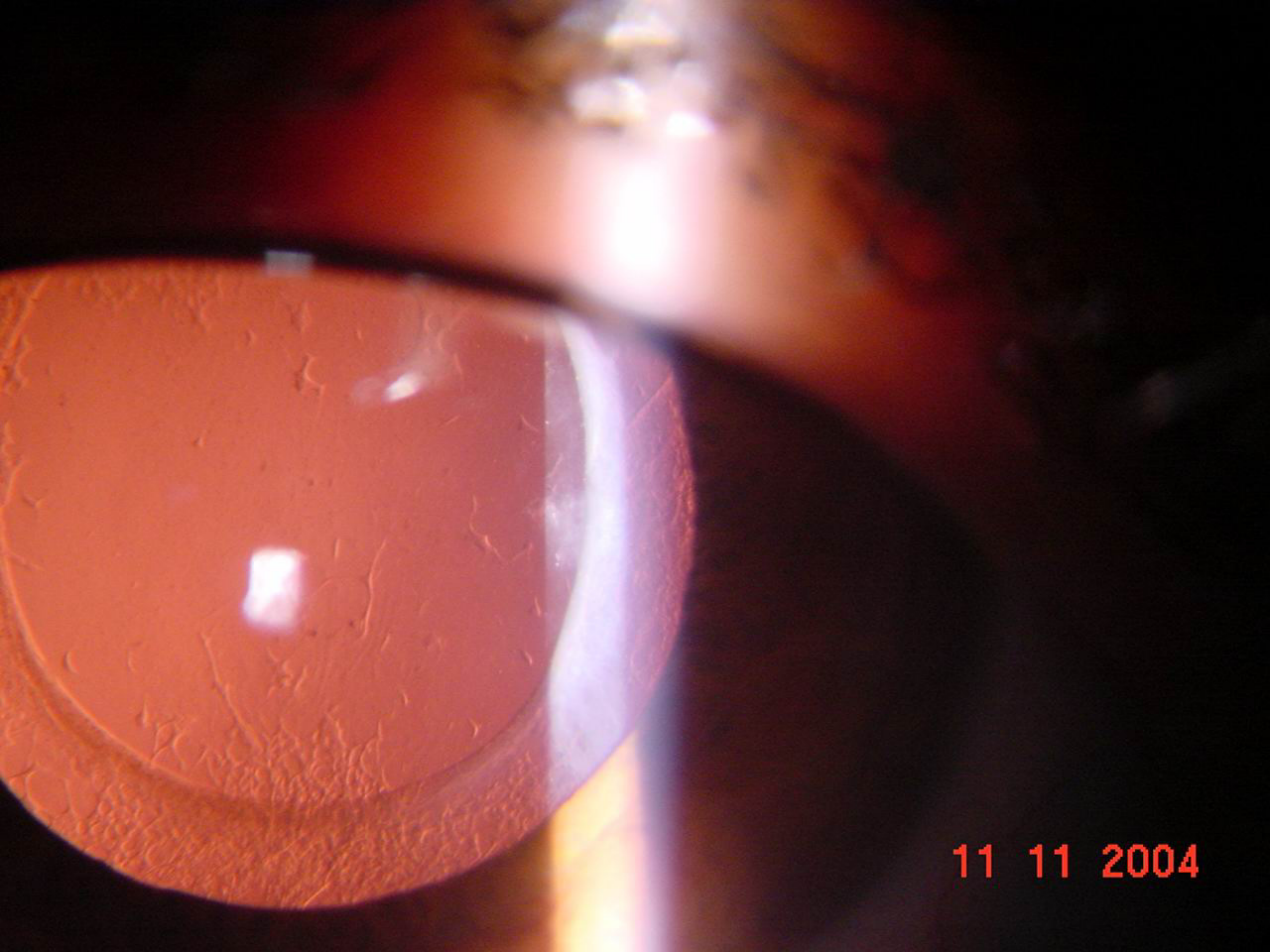
Resolution of anterior uveitis: Slit lamp examination showing slight opacification of the posterior capsule.

**Figure 3:**
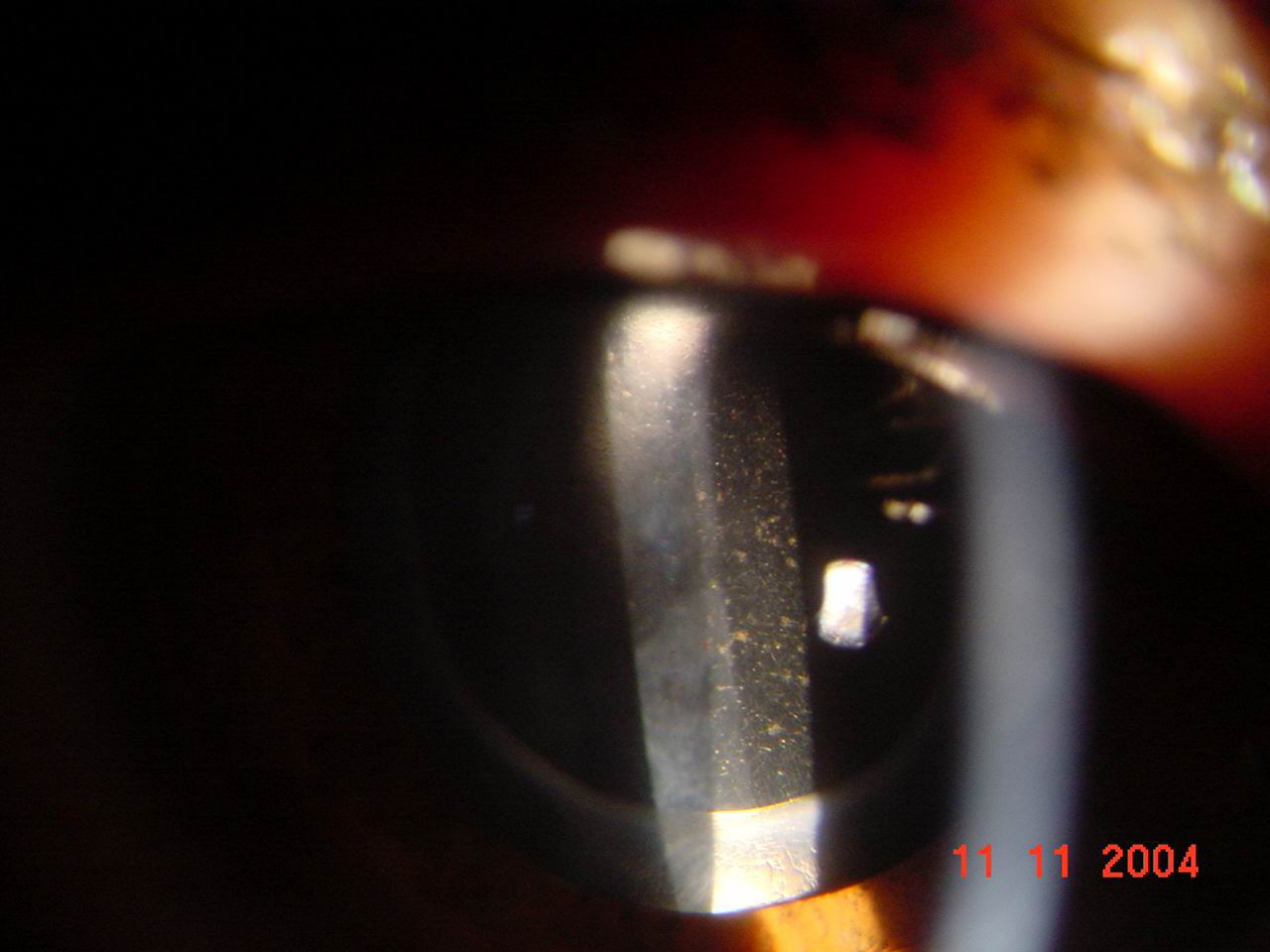
Resolution of anterior uveitis: Slit lamp examination showing residual pigmented deposits on the surface of the intraocular lens.

The patient was scheduled to have zoledronic acid 4 mg once every six months for osteoporosis. In August 2004, the patient was given intravenous hydration with normal saline, premedicated with paracetamol, and had an intravenous infusion of zoledronic acid 4 mg in 100 cc normal saline given over 30 minutes. The patient did well throughout the infusion; however, she felt chilly and feverish overnight. The next day, she presented to the Emergency Department complaining of severe right eye pain and decreased vision. Examination revealed uncorrected visual acuity of 20/100 in the right eye and 20/60 in the left eye. The intraocular pressure was 14 mmHg in both eyes. Slit lamp examination revealed marked ciliary injection of the right eye, and a 2+ flare and inflammatory cells in the anterior chamber. An inflammatory fibrinous pupillary membrane was also present (Fig 1). Fundus examination was normal. A diagnosis of right anterior uveitis was made. The patient was placed on topical prednisone every 6 hours, and atropine eyedrops every 12 hours. She responded well to the treatment which was gradually tapered off over a period of 6 weeks without recurrence of uveitis. Following treatment, visual acuity in the right eye improved to 20/30, and slit lamp examination revealed slight opacification of the posterior capsule (Fig 2), and residual pigmented deposits on the surface of the intraocular lens (Fig 3). These inflammatory changes and deposits on the posterior lens surface disappeared on their own over a period of 3 months, but residual posterior capsular opacification (PCO) is still present. This PCO has no effect on the patient's visual acuity at present and doesn't require any intervention.

Unlike in metastatic bone diseases where bisphosphonate therapy is administered on a monthly basis, our plan was to give the patient zoledronic acid every six months [11]. The patient refused to have any further bisphosphonate therapy

## Conclusion

This is the first report of unilateral anterior uveitis occuring within 24 hours of zoledronic acid in a patient with an implanted intraocular lens. The clinical symptoms were severe and dramatic but the ophtalmologic examination showed a fibrinous anterior uveitis of moderate severity. The patient gradually responded well to treatment and recovered after three months with no major sequalae. The inflammatory deposits on the posterior lens surface disappeared over a period of 3 months, except for residual PCO which is a common occurrence after cataract surgery and intraocular inflammation [12].

Zoledronic acid is the most widely used bisphosphonate for metastatic bone disease and osteoporosis because of its relative higher potency and short infusion time. Every internist, oncologist, endocrinologist, and ophtalmologist should be aware of uveitis as a possible complication of zoledronic acid. Careful review of systems should include ophtalmologic history. Patients should be instructed to immediately report to their physicians any eye complaints. Treatment with topical prednisone and atropine eyedrops should be instituted immediately at the onset of symptoms. Patients should be instructed to consult their ophthalmologist for any ocular complaints. Close follow-up of these patients should also be made. This report documents anterior uveitis as a complication of zoledronic acid therapy. This side effect could be an idiosyncratic reaction but research into etiology may shed more light on mechanisms of action of bisphosphonates and interaction with lenses, if any.

Repeating the administration of zoledronic acid in these patients with prophylactic topical steroids and atropine remains an unresolved issue. In one case report of uveitis associated with clodronate, the patient developed the same ocular symptoms when rechallenged with the same drug [13].

## Competing interests

The author(s) declare that they have no competing interests.

## Authors' contributions

**NSES **was the main operator in charge of the case, the idea and the manuscript.

**ZKO **participated in the literature search and manuscript formatting.

**JHB **managed the uveitis, prepared the figures, and opthalmology descriptions..

All authors contributed to the preparation of the manuscript and literature review.

All authors read and approved the final version of the manuscript.

## Pre-publication history

The pre-publication history for this paper can be accessed here:



## References

[B1] Robertson WG, Morgan DB, Fleisch H, Francis MD (1971). The effects of diphosphonates on the exchangeable and non-exchangeable calcium and phosphate of hydroxyapatite. Biochim Biophys Acta.

[B2] Conte P, Guarneri V (2004). Safety of intravenous and oral bisphosphonates and compliance with dosing regimens. Oncologist.

[B3] Mbekeani JN, Slamovits TL, Schwartz BH, Sauer HL (1999). Ocular inflammation associated with alendronate therapy. Arch Ophthalmol.

[B4] Salmen S, Berrueta L, Sanchez N, Montes H, Borges L (2002). Nongranulomatous anterior uveitis associated with alendronate therapy. Invest Clin.

[B5] Malik AR, Campbell SH, Toma NM (2002). Bilateral acute anterior uveitis after alendronate. Br J Ophthalmol.

[B6] Ghose K, Waterworth R, Trolove P, Highton J (1994). Uveitis associated with pamidronate. Aust N Z J Med.

[B7] Rey J, Daumen-Legre V, Pham T, Bernard P, Dahan L, Acquaviva PC, Lafforgue P (2000). Uveitis, an under-recognized adverse effect of pamidronate. Case report and literature review. Joint Bone Spine.

[B8] Fraunfelder FW, Fraunfelder FT, Jensvold B (2003). Scleritis and other ocular side effects associated with pamidronate disodium. Am J Ophthalmol.

[B9] Durnian JM, Olujohungbe A, Kyle G (2005). Bilateral acute uveitis and conjunctivitis after zoledronic acid therapy. Eye.

[B10] Goss PE, Ingle JN, Martino S, Robert NJ, Muss HB, Piccart MJ, Castiglione M, Tu D, Shepherd LE, Pritchard KI, Livingston RB, Davidson NE, Norton L, Perez EA, Abrams JS, Therasse P, Palmer MJ, Pater JL (2003). A randomized trial of letrozole in postmenopausal women after five years of tamoxifen therapy for early-stage breast cancer. N Engl J Med.

[B11] Reid IR (2003). Bisphosphonates: new indications and methods of administration:. Curr Opin Rheumatol.

[B12] Emery J (1999). Capsular opacification after cataract surgery:. Curr Opin Ophthalmol.

[B13] Fietta P, Manganelli P, Lodigiani L (2003). Clodronate induced uveitis. Ann Rheum Dis.

